# Supplementary figures and images for: Genome-wide association study of early liveweight traits in fat-tailed Akkaraman lambs
Source: PLoS One. 2023 Nov 21;18(11):e0291805. doi: 10.1371/journal.pone.0291805 (PMC10662757; doi:10.1371/journal.pone.0291805)

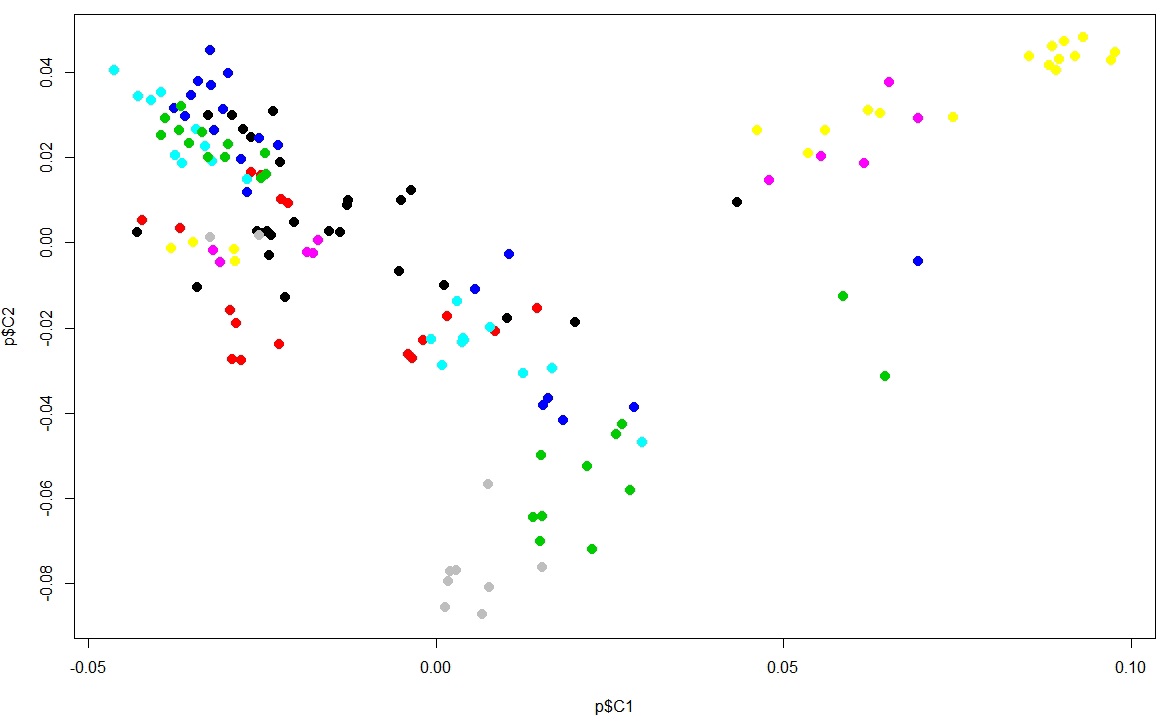

Supplement: S1 Fig — (JPG) [file pone.0291805.s001.jpg]

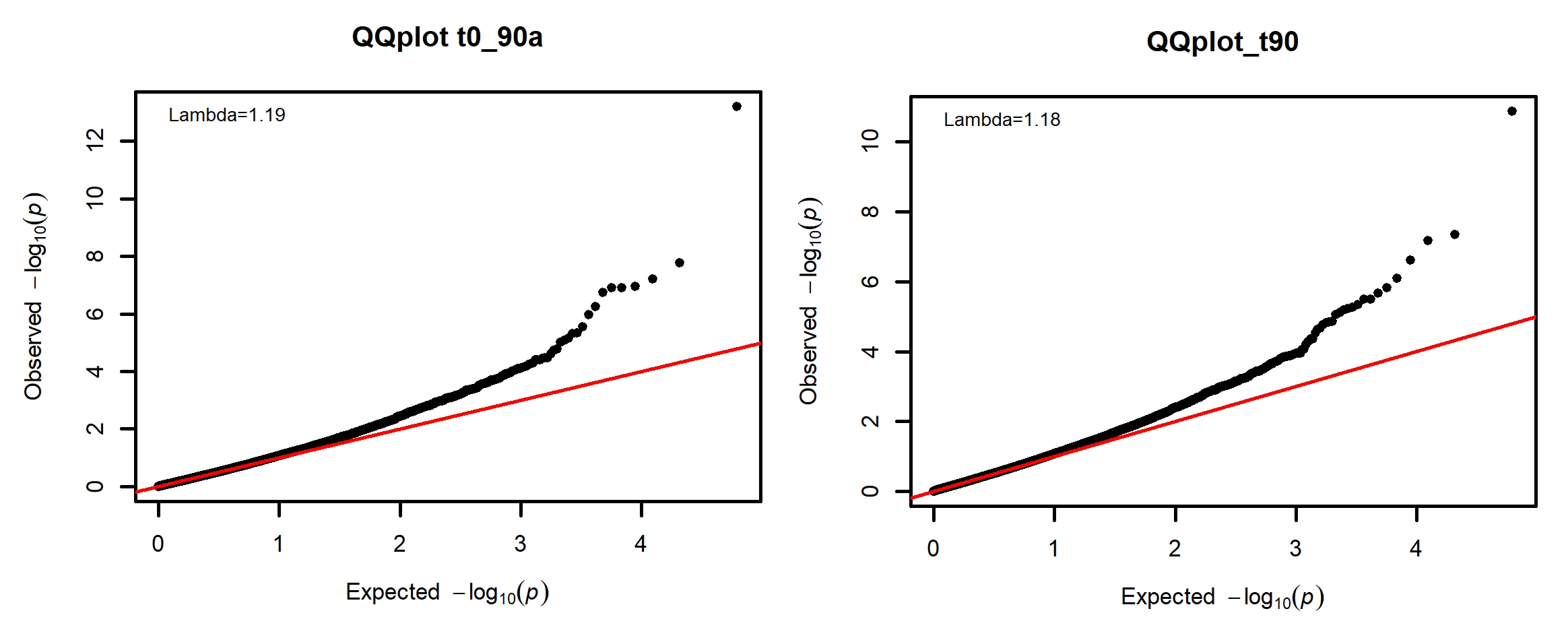

Supplement: S2 Fig — The x-axis and y-axis represent −log10 transformed expected p-values and observed p-values, respectively. The dots indicate −log10(p) of the SNPs and the diagonal line represents the expected values under the null hypothesis for no association. (JPG) [file pone.0291805.s002.jpg]
