# Supplementary material for: Genome-wide association study of early liveweight traits in fat-tailed Akkaraman lambs
Source: PLoS One. 2023 Nov 21;18(11):e0291805. doi: 10.1371/journal.pone.0291805 (PMC10662757; doi:10.1371/journal.pone.0291805)
Supplement: S1 Table — (DOCX) [file pone.0291805.s003.docx]

**Supporting Information:**

**S1** **Table.** Measured liveweights and average daily gain values for Akkaraman lambs (in kg).

| **Variable** | **N** | **Mean** | **Std Dev** | **Minimum** | **Maximum** |
| --- | --- | --- | --- | --- | --- |
| Birth weight | 194 | 4.74 | 0.84 | 2.25 | 6.79 |
| D30 | 194 | 11.19 | 2.17 | 6.78 | 17.30 |
| D60 | 194 | 18.70 | 3.90 | 11.45 | 28.61 |
| D90 | 192 | 27.92 | 5.44 | 16.26 | 41.46 |
| ADG30 | 194 | 0.37 | 0.07 | 0.22 | 0.57 |
| ADG60 | 194 | 0.31 | 0.06 | 0.19 | 0.47 |
| ADG90 | 192 | 0.31 | 0.06 | 0.18 | 0.46 |
| ADG30_60 | 194 | 0.25 | 0.07 | 0.07 | 0.46 |
| ADG60_90 | 192 | 0.30 | 0.08 | 0.04 | 0.54 |
| ADG30_90 | 192 | 0.28 | 0.07 | 0.08 | 0.43 |
